# Comparative Study to Evaluate the Accuracy of Differential Diagnosis Lists Generated by Gemini Advanced, Gemini, and Bard for a Case Report Series Analysis: Cross-Sectional Study

**DOI:** 10.2196/63010

**Published:** 2024-10-02

**Authors:** Takanobu Hirosawa, Yukinori Harada, Kazuki Tokumasu, Takahiro Ito, Tomoharu Suzuki, Taro Shimizu

**Affiliations:** 1 Department of Diagnostic and Generalist Medicine Dokkyo Medical University Shimotsuga Japan; 2 Department of General Medicine Graduate School of Medicine, Dentistry and Pharmaceutical Sciences Okayama University Okayama Japan; 3 Satsuki Home Clinic Tochigi Japan; 4 Department of Hospital Medicine Urasoe General Hospital Okinawa Japan

**Keywords:** artificial intelligence, clinical decision support, diagnostic excellence, generative artificial intelligence, large language models, natural language processing

## Abstract

**Background:**

Generative artificial intelligence (GAI) systems by Google have recently been updated from Bard to Gemini and Gemini Advanced as of December 2023. Gemini is a basic, free-to-use model after a user’s login, while Gemini Advanced operates on a more advanced model requiring a fee-based subscription. These systems have the potential to enhance medical diagnostics. However, the impact of these updates on comprehensive diagnostic accuracy remains unknown.

**Objective:**

This study aimed to compare the accuracy of the differential diagnosis lists generated by Gemini Advanced, Gemini, and Bard across comprehensive medical fields using case report series.

**Methods:**

We identified a case report series with relevant final diagnoses published in the *American Journal Case Reports* from January 2022 to March 2023. After excluding nondiagnostic cases and patients aged 10 years and younger, we included the remaining case reports. After refining the case parts as case descriptions, we input the same case descriptions into Gemini Advanced, Gemini, and Bard to generate the top 10 differential diagnosis lists. In total, 2 expert physicians independently evaluated whether the final diagnosis was included in the lists and its ranking. Any discrepancies were resolved by another expert physician. Bonferroni correction was applied to adjust the *P* values for the number of comparisons among 3 GAI systems, setting the corrected significance level at *P* value <.02.

**Results:**

In total, 392 case reports were included. The inclusion rates of the final diagnosis within the top 10 differential diagnosis lists were 73% (286/392) for Gemini Advanced, 76.5% (300/392) for Gemini, and 68.6% (269/392) for Bard. The top diagnoses matched the final diagnoses in 31.6% (124/392) for Gemini Advanced, 42.6% (167/392) for Gemini, and 31.4% (123/392) for Bard. Gemini demonstrated higher diagnostic accuracy than Bard both within the top 10 differential diagnosis lists (*P*=.02) and as the top diagnosis (*P*=.001). In addition, Gemini Advanced achieved significantly lower accuracy than Gemini in identifying the most probable diagnosis (*P*=.002).

**Conclusions:**

The results of this study suggest that Gemini outperformed Bard in diagnostic accuracy following the model update. However, Gemini Advanced requires further refinement to optimize its performance for future artificial intelligence–enhanced diagnostics. These findings should be interpreted cautiously and considered primarily for research purposes, as these GAI systems have not been adjusted for medical diagnostics nor approved for clinical use.

## Introduction

### Diagnostic Team to Reduce Misdiagnoses

Diagnosis is a crucial step in clinical medicine, where a significant proportion of medical errors and harms are related to diagnostic errors [1]. The formation of a diagnostic team has been proposed as an effective strategy to mitigate the risks associated with misdiagnosis [2,3]. This team should promote collaboration among medical professionals, patients, and their families, and the integration of digital tools to enhance diagnostic accuracy [4]. Several research, including systematic reviews, have shown that the implementation of clinical decision support systems (CDSSs) in clinical settings has significantly improved diagnostic accuracy, patient care, and health care process [5-7].

### Digital Tool for Medical Diagnosis

Various digital tools, particularly diagnostic CDSSs, have emerged for medical diagnostics. These systems are designed to provide diagnostic suggestions based on clinical data, aiding medical professionals in clinical decision-making [8]. Traditionally, diagnostic CDSSs, such as symptom checkers and differential diagnosis generators, have relied on fixed algorithms and rule-based systems derived from medical databases and expert input [9-11]. Unfortunately, these systems often experience poor accuracy and inadequate integration into clinical workflows, limiting their practical use in real-world medical settings [4]. In this context, artificial intelligence (AI), especially generative AI (GAI), has introduced a new category of CDSS [12]. This advancement suggests a future shift in how digital tools can support diagnostic processes.

### GAI in Medical Diagnosis

GAI systems have shown rapid development and are increasingly influencing various fields, including medicine. This advancement is partly due to the development of machine learning techniques, such as neural networks and natural language processing. GAI represents a shift from rule-based systems to models that can autonomously generate and evaluate new data patterns. Overcoming many limitations faced by traditional CDSSs, GAI systems could significantly enhance future diagnostic processes. Notable examples include ChatGPT developed by Open AI, and Gemini and Gemini Advanced from Google [13]. These systems use advanced large language models (LLMs), which are complex neural networks trained on vast data sets through natural language processing [14]. Recent studies, including one evaluating dermoscopy image descriptions with chatbot responses, have demonstrated promising results in accuracy and diagnostic completeness by ChatGPT [15]. The language model for dialogue applications (LaMDA) developed by Google AI is one such LLM. Their ability to process and generate outputs is particularly promising for future applications in medical diagnostics, where they will analyze complex clinical information and collaborate as part of a diagnostic team.

### From Bard to Gemini and Gemini Advanced

Originally, Bard was developed using the LaMDA model primarily for text generation and conversational AI and later transitioned to the Pathways Language Model (Palm 2). Subsequent developments led to the release of Gemini and Gemini Advanced in December 2023. Gemini Advanced, an upgraded version of Gemini, leverages Ultra 1.0, Google’s most advanced model, offered as a fee-based service [16,17]. These developments reflect the rapid pace at which GAI technology is advancing. Recent updates have transformed Bard into Gemini and Gemini Advanced, enhancing their functionalities and applications in various fields. Previous research, including our own, has demonstrated that Bard showed promising results in medicine [18-21]. Moreover, a recent study has shown that several GAI systems, including Gemini Advanced, could achieve notable diagnostic accuracy for multiple-choice questions about clinical vignettes [22]. These findings suggest that even without specific training or reinforcement for diagnostics, GAI systems show potential for reliable use in diagnostics. Despite these advancements, the comparative diagnostic accuracy of differential diagnosis lists by these GAI systems across comprehensive medical fields remains to be fully explored. This study aims to fill that gap by evaluating the diagnostic accuracy of the differential diagnosis lists generated by Gemini Advanced, Gemini, and Bard for case report series across various medical disciplines.

## Methods

### Overview

**Figure 1:**
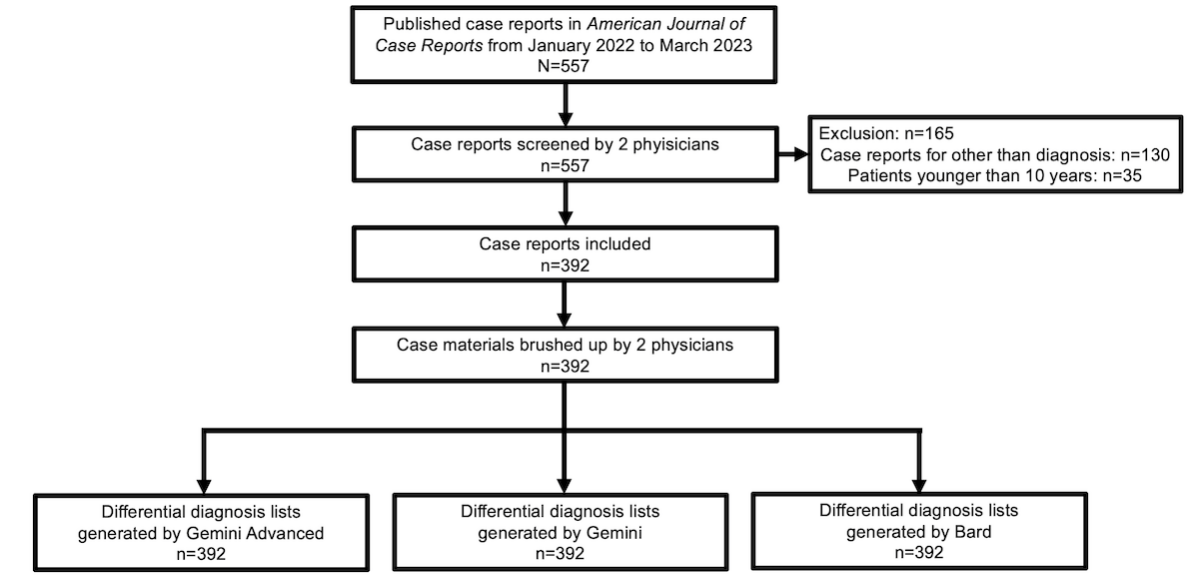
Study flow of inclusion case materials and generation of differential diagnosis.

An experimental study was conducted to assess the diagnostic accuracy of Gemini Advanced, Gemini, and Bard for a comprehensive case report series. This study was conducted at the Department of Diagnostic and Generalist Medicine (General Internal Medicine) at Dokkyo Medical University, Japan. This study consisted of preparing case materials, generating differential diagnosis lists, evaluating the lists, and analyzing the diagnostic accuracy. Figure 1 shows the study flow, including the inclusion of case materials and the generation of differential diagnosis lists.

### Preparing Case Materials

We focused on a comprehensive series of case reports from the *American Journal of Case Reports*, covering a broad range of medical fields. The structured format of the journal facilitated easy identification of sections containing the case reports and the final diagnoses. Initially, the inclusion criteria were the case reports published in the *American Journal of Case Reports* from January 1, 2022, to March 1, 2023. A PubMed search identified 557 consecutive case reports. After excluding 130 nondiagnostic case reports and 35 pediatric case reports (patients aged 10 years and younger), 392 case reports remained. The exclusion criteria were based on previous research for CDSS [23]. We refined the case reports to prepare the case materials, which typically included the initial case report part to the definitive tests for final diagnosis. The relevant final diagnoses were typically described by the authors. We used only textual data exclusively, omitting image data. Specifically, the title, background, final diagnosis, clinical course following diagnosis, discussion, conclusion, figures, tables, and supplemental materials were excluded from the case materials. The main investigator (TH) conducted this process with validation from another investigator (YH). The PubMed search keywords are shown in Multimedia Appendix 1. For example, in a case report titled “Herpes Zoster Following COVID-19 Vaccine Booster,” the final diagnosis was herpes zoster [24]. We extracted the case report part from “An 82-year-old..” to “Vesicular breath sounds were heard equally on both lung fields.”

### Generating Differential Diagnosis Lists

We used Gemini Advanced, Gemini, and Bard as GAI systems for this research. This was because these systems are popular AI platforms available to the public. These GAI systems were not specifically enhanced for medical diagnosis. Details about the GAI systems used in this study are provided in Table 1. To generate the top 10 differential diagnosis lists from GAI systems, the main investigator typically copied and pasted the case materials into the AI systems with the prompt, that is “Tell me the top 10 suspected illnesses for the following case: (case materials).” This prompt, developed through preliminary research, aimed to generate the top 10 differential diagnosis lists. The first list produced by the GAI systems was used as the differential diagnosis list. The data control setting was adjusted to “Not saving activity,” to avoid the influence from the previous conversations. In addition, before starting a new session, the main investigator refreshed the previous session to prevent any influence from previous conversations.

**Table 1:** The details of generative artificial intelligence systems used in this study.

Gemini Advanced		Gemini	Bard
**AI^a^ model**			
	Ultra 1.0	Pro	Pathways Language Model (Palm 2)-based
**Availability**			
	Fee-based subscription	Free with user login	Discontinued
**The setting of the app activity**			
	Not saving activity	Not saving activity	Not saving activity
**Access date**			
	April 4-9, 2024	March 12-28, 2024	July 1, 2023-August 8 2023
**Prompt**			
	“Tell me the top 10 suspected illnesses for the following case: (case materials).”	“Tell me the top 10 suspected illnesses for the following case: (case materials).”	“Tell me the top 10 suspected illnesses for the following case: (case materials).”

### Evaluating the Differential Diagnosis Lists

A total of 2 expert researchers (TI and T Suzuki) independently evaluated the differential diagnosis lists from GAI systems. A score of “1” was assigned if the differential accurately and specifically identified the final diagnosis or was sufficiently close to the final diagnosis. Conversely, a score of “0” was assigned if it diverged significantly from the final diagnosis [25]. When a GAI system could not output the differential diagnosis list, a score of “0” was labeled. When the score was “1,” the evaluator assessed its ranking within the list. Any discrepancies were resolved by another expert researcher (KT). All evaluators were blinded to which GAI systems produced the differential diagnosis lists.

### Analyzing the Diagnostic Accuracy

In this study, we defined diagnostic accuracy as the inclusion of the final diagnoses in the differential diagnosis lists.

### Outcome

In terms of the outcomes, the primary outcome was the total score for correctly identifying the final diagnosis in the top 10 differential diagnosis lists generated by Gemini Advanced, Gemini, and Bard. The total number of included case reports was used as the denominator. The numerator was the number of case reports that correctly identified the final diagnosis in the top 10 differential diagnosis lists. The secondary outcomes were the total score for correctly identifying the final diagnosis in the top 5 differential diagnosis lists and as the top diagnosis generated from Gemini Advanced, Gemini, and Bard.

In addition, we evaluated the top 10 rankings of the most frequently named differential diagnoses across generated differential diagnosis lists by a GAI system to find the underlying patterns. We also assessed whether the items in the differential diagnosis lists corresponded to the names of existing diseases.

Moreover, we analyzed how Gemini Advanced, Gemini, and Bard rank the correct diagnosis on average when it appears in the differential diagnosis lists. This metric helps evaluate not only whether the correct diagnosis is included but also its relative priority among other suggested diagnoses. For cases where the correct diagnosis was missing, we assigned a penalty rank; specifically, we used 11 as the penalty rank.

### Statistical Analysis

A chi-square test was used for the categorical or binary variables. The Mann-Whitney *U* test was applied to analyze the average rankings. For multiple comparisons, the Bonferroni correction was applied [26]. The Bonferroni correction adjusts the *P* value by dividing it by the number of comparisons being made, namely 3 GAI systems, that consist of Gemini Advanced, Gemini, and Bard, setting a more stringent threshold for individual tests to be considered significant. The Bonferroni-corrected significance level was defined as a *P* value of <.02. Note that all statistical tests were performed using the R (version 4.2.2; The R Foundation for Statistical Computing) for macOS X.

### Ethical Considerations

Given the study’s method of using published case reports, approval from an ethical committee was deemed not applicable.

## Results

### Overview of Outputs From GAI Systems

In total, 392 case reports were included. The distribution of diagnoses in these reports highlighted the prevalence of complex medical conditions, with 22.7% (89/392) of cases being diagnosed as malignancy, 22.4% (88/392) as various infections, and 17.1% (67/392) as vascular diseases.

Bard generated differential diagnosis lists for all 392 cases. In contrast, both Gemini Advanced and Gemini encountered challenges with 3 cases, failing to produce outputs. The cases where outputs were not generated involved complex clinical scenarios that were directly described in the reports as involving overdose, psychological conditions, and vaccination side effects. Representative outputs from Gemini Advanced, Gemini, and Bard are shown in Table 2. The data set of differential diagnoses generated by AI systems in this study, alongside the final diagnosis, are shown in Multimedia Appendix 2.

**Table 2:** Representative outputs from Gemini Advanced, Gemini, and Bard for final diagnoses.

Gemini Advanced		Gemini	Bard
**Herpes Zoster**			
	Shingles (Herpes Zoster) Myocardial ischemia Costochondritis Pericarditis Pleuritis Pulmonary embolism Pneumonia Metastatic malignancy Aortic dissection Esophageal spasm	Shingles (Herpes Zoster) Myocardial ischemia (Angina) Costochondritis Musculoskeletal strain Pleurisy Intercostal neuritis Pulmonary embolism Pericarditis Gastritis Esophagitis	Herpes zoster (Shingles) Myocardial infarction (heart attack) Aortic dissection Pulmonary embolism Pneumonia Thoracic radiculopathy Costochondritis Intercostal neuralgia Costoclavicular syndrome Tietze syndrome
**Diffuse alveolar hemorrhage**			
	Pulmonary alveolar hemorrhage Nocardia pneumonia Pulmonary embolism Acute eosinophilic pneumonia Drug-induced pneumonitis Legionnaires' disease Hypersensitivity pneumonitis Idiopathic pulmonary fibrosis Bronchiolitis obliterans organizing pneumonia Acute interstitial pneumonitis	Pulmonary alveolar hemorrhage Trastuzumab-induced pneumonitis Aspirin-exacerbated respiratory disease Acute heart failure Pulmonary edema Nocardia pneumonia Pulmonary hypertension Acute interstitial lung disease Acute chronic kidney disease Occult gastrointestinal bleed	Acute radiation pneumonitis Acute hypersensitivity pneumonitis Pulmonary edema Pulmonary embolism Aspiration pneumonia Bronchogenic carcinoma Sarcoidosis Lymphoma Wegener’s granulomatosis Goodpasture syndrome
**Glomus tumor**			
	Meralgia paresthetica Soft tissue sarcoma Fibroma Myositis ossificans Neuroma Hemangioma Liposarcoma Lymphoma Abscess Malignant peripheral nerve sheath tumor	Meralgia paresthetica Lumbar radiculopathy Femoral neuropathy Piriformis syndrome Muscle strain Myofascial pain syndrome Glomus tumor Neuroma Soft tissue sarcoma (unlikely) Abscess (unlikely)	Lipoma Fibromatosis Myositis Neoplasm Infection Inflammation Trauma Hematoma Varicose vein Cellulitis

### Diagnostic Accuracy

**Figure 2:**
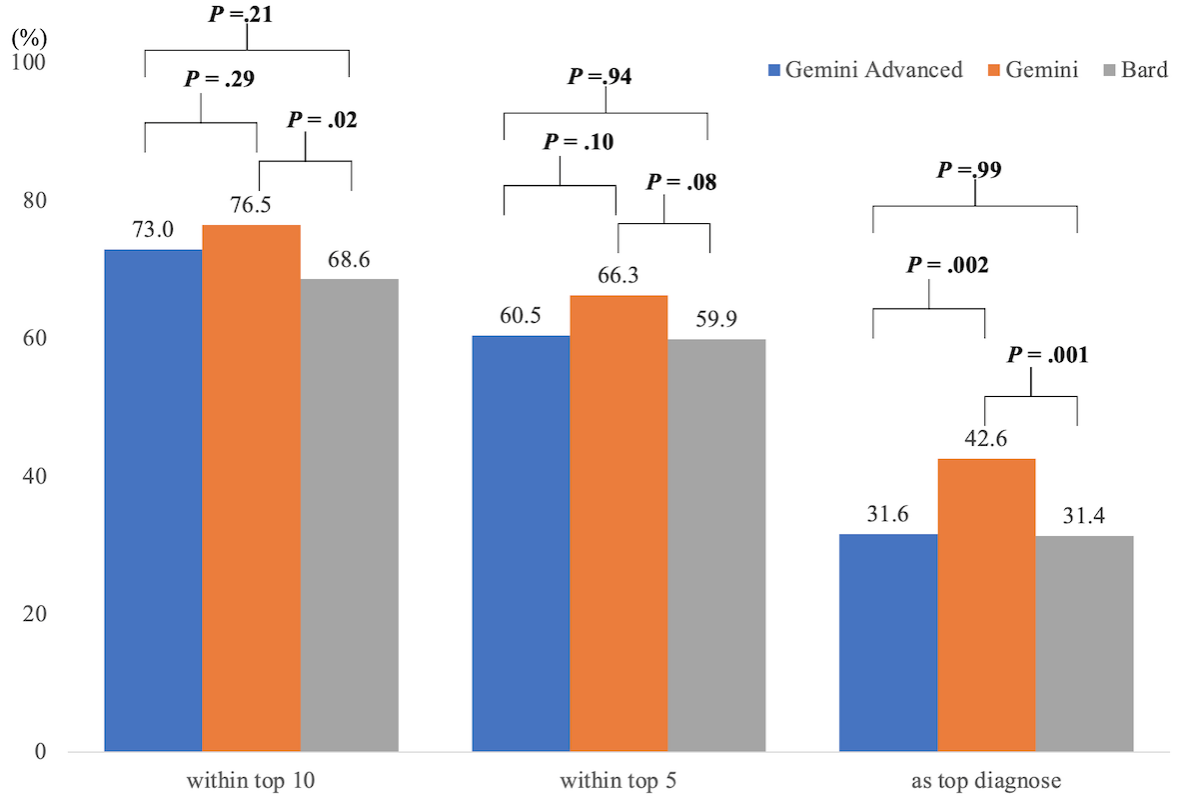
Diagnostic accuracy of Gemini Advanced, Gemini, and Bard. *P* values were derived from the chi-square test. The Bonferroni-corrected significance level at a *P* value <.02.

In terms of diagnostic accuracy, the inclusion rates of the final diagnoses within the top 10 differential diagnosis lists were 73.0% (286/392) for Gemini Advanced, 76.5% (300/392) for Gemini, and 68.6% (269/392) for Bard. For the top 5 differential diagnoses, the rates were 60.5% (237/392) for Gemini Advanced, 66.3% (260/392) for Gemini, and 59.9% (235/392) for Bard. The top diagnoses matched the final diagnoses in 31.6% (124/392) for Gemini Advanced, 42.6% (167/392) for Gemini, and 31.4% (123/392) for Bard. Gemini demonstrated higher diagnostic accuracy than Bard both within the top 10 differential diagnosis lists (*P*=.02) and as the top diagnosis (*P*=.001). In addition, Gemini Advanced achieved lower accuracy in identifying the most probable diagnosis, compared with Gemini with this result being statistically significant (*P*=.002). Other comparisons were statistically insignificant. Table 3 and Figure 2 show the diagnostic accuracy by Gemini Advanced, Gemini, and Bard.

**Table 3:** Diagnostic accuracy of Gemini Advanced, Gemini, and Bard.

Variable	Gemini Advanced (N=392), n (%)	Gemini (N=392), n (%)	Bard (N=392), n (%)	*P* value^a^		
				Gemini Advanced versus Gemini	Gemini Advanced versus Bard	Gemini versus Bard
Within the top 10	286 (73.0)	300 (76.5)	269 (68.6)	.29	.21	.02
Within the top 5	237 (60.5)	260 (66.3)	235 (59.9)	.10	.94	.08
Top diagnosis	124 (31.6)	167 (42.6)	123 (31.4)	.002	.99	.001

^a^Chi-square test. The Bonferroni-corrected significance level at a *P* value <.02.

### Most Frequently Named Differential Diagnoses

Regarding the top 10 most frequently named differential diagnoses, all rankings included sepsis, pneumonia, pulmonary embolism, lymphoma, and meningitis. Notably, the top 3 most frequently named differential diagnoses by Gemini Advanced and Gemini were the same. Table 4 shows the top 10 most frequently named differential diagnoses generated by Gemini Advanced, Gemini, and Bard.

**Table 4:** The top 10 most frequently named differential diagnoses were generated by Gemini Advanced, Gemini, and Bard.

The raking in the top 10 most frequently named differentials, (N)	Gemini Advanced (n)	Gemini (n)	Bard (n)
1	Sepsis (43)	Sepsis (42)	Sarcoidosis (51)
2	Pneumonia (34)	Pneumonia (28)	Sepsis (42)
3	Pulmonary embolism (33)	Pulmonary embolism (20)	Pneumonia (41)
4	Acute kidney injury (28)	Sarcoidosis (15)	Lymphoma (40)
5	Lymphoma (25)	Pericarditis (14)	Pulmonary embolism (39)
6	Urinary tract infection (24)	Meningitis (14)	Meningitis (31)
7	Heart failure (23)	Lymphoma (13)	Inflammatory bowel disease (29)
8	Meningitis (22)	Myocarditis (13)	Tuberculosis (26)
9	Myocardial infarction (20)	Acute kidney injury (12)	Encephalitis (25)
10	Pericarditis (18)	Systemic lupus erythematosus (12)	Myocarditis (24)

### Inappropriate Diseases Names

From all differential diagnosis lists output by generative AIs, we identified inappropriate disease names: 11 items from Gemini Advanced, 9 items from Gemini, and 5 items from Bard. Notably, Gemini Advanced and Gemini both erroneously listed “Wegner’s granulomatosis,” a misspelling of the previous correct term, “Wegener’s granulomatosis,” which has now been updated to “Granulomatosis with Polyangiitis” [27]. Another error by Gemini Advanced involved “Microcytic colitis,” likely a confusion between “microcystic anemia” and “microscopic colitis.” Table 5 lists the inappropriate disease names generated by Gemini Advanced, Gemini, and Bard.

**Table 5:** Inappropriate disease name generated by Gemini Advanced, Gemini, and Bard.

Correct disease name, [cell number in Multimedia Appendix 2]	Inappropriate disease name by Gemini Advanced	Inappropriate disease name by Gemini	Inappropriate disease name by Bard
Drug reaction (Dasatinib)	Drug reation (Dasatinib) [O15]	—^a^	—
Nonketotic hyperglycemic hyperosmolar coma	—	Nonketotic hyperglycemia hyperosmolar coma [X29]	—
Lipedema	—	Lipoderma [U34]	—
Small bowel angiodysplasia	Small bowel angiodisplasia [M40]		—
Granulomatosis with polyangiitis	Granulomatous with polyangiitis (L68), Wegner’s granulomatosis [N111]	Wegner’s granulomatosis [U186]	—
Costochondritis	Costochondritisa [P86]	—	—
Maxillary sinus carcinoma	—	Maxillary sinus cycinoma [L106]	—
Constrictive pericarditis	Conrictive pericarditis [L110]	—	—
Scleroderma-related interstitial lung disease	—	Scleroderma-related interstitial lung disease [S117]	—
Pericoronitis	—	—	Pericoronatitis [AA133]
Osteitis	—	—	Osteoitis [AC133]
Microscopic colitis	Microcytic colitis [L152]	—	—
Pneumocystis jirovecii	—	—	Pneumocystis jerovecii [AG156]
Leukoencephalopathy	—	Leukoencephalomyopathy [X195]	—
Strumal carcinoid	—	Struma carcinoid [W208]	—
Restrictive ventilatory impairment	Restricted ventilatory impairment [J360]	—	—
Moebius syndrome	—	Mobius syndrome [Z369]	—
Endometriosis	—	—	Endometrios [AE385]
Cryptococcus neoformans	—	—	Chryptococcus neoformans [AJ389]
Unknown	Ytzinger hernia [M197]	Y-type appendicitis [V197]	—
Unknown	(There was partly Arabic language) [L292]	—	—
Unknown (Transaminase elevation is also not disease name)	Transaminitis elevation (N354)	—	—

^a^Not applicable.

### Average Ranking

In terms of average ranking, the scores were 5.25 (SD 4.16) for Gemini Advanced, 4.54 (SD 4.21) for Gemini, and 5.33 (SD 4.29) for Bard. The differences in average rankings were not statistically significant between Gemini Advanced and Gemini (*P*=.99), between Gemini Advanced and Bard (*P*=.17), and between Gemini and Bard (*P*=.99).

## Discussion

### Principal Findings

In the following, we discuss our principal findings. Our findings indicate that Gemini demonstrated superior diagnostic accuracy compared with Bard, not only within the top 10 differential diagnosis lists but also in identifying the most likely diagnosis. Specifically, Gemini’s diagnostic accuracy for the top 10 lists was 76.5% (300/392), compared to Bard’s 68.6% (269/392), with a statistically significant difference (*P*=.02). Moreover, as the top diagnosis, Gemini’s diagnostic accuracy was 42.6% (167/392) versus Bard’s 31.4% (123/392), also significant (*P*=.001). This enhancement in Gemini’s diagnostic performance may be attributed to its advanced algorithmic framework, which likely incorporates more nuanced medical data and learns from recent case inputs, leading to more refined diagnostic predictions.

However, the performance of Gemini did not statistically outperform in the top 5 differential diagnosis lists. This outcome may suggest that while Gemini’s algorithm is effective in a broader exploratory context, its precision may falter when constrained to a narrower list of top diagnoses. This indicates that a balance between breadth of exploration and depth of focus is crucial for optimizing diagnostic accuracy in such AI systems.

Conversely, our analysis showed that Gemini Advanced did not perform as well as expected when compared with Gemini. Despite expectations that the advanced model would provide enhanced diagnostic capabilities, it achieved lower accuracy in identifying the most probable diagnosis with 31.6% (124/392) compared to Gemini’s 42.6% (167/392), with this result being statistically significant (*P*=.002). This outcome suggests that the additional features or complexity added in Gemini Advanced may not necessarily translate into improved diagnostic performance. These findings underscore the need for further refinement and optimization of Gemini Advanced to harness its potential for future AI-enhanced diagnostics.

In addition, our analysis identified issues with inappropriate disease naming in the outputs from GAI systems, with Gemini Advanced and Gemini producing outdated or misspelled terms for vasculitis, instead of using the updated name. These inaccuracies highlight the challenges in ensuring up-to-date and precise medical terminology in AI outputs, which is crucial for maintaining trust and reliability in AI-assisted diagnostics. Furthermore, these misspellings are often found in published medical articles, suggesting that GAIs may have learned these errors from these sources. The fact that both Gemini Advanced and Gemini exhibited the same mistakes indicates potential similarities in their underlying models or training data.

Regarding average rankings, there were no statistically significant differences among generative AI systems. This indicates a level of parity in how each model ranks diagnoses when they include the correct diagnosis, suggesting that while there are differences in overall accuracy, the ranking mechanisms of each model are relatively similar.

Given the current performance metrics, our analysis supports prioritizing the adjustment and enhancement of Gemini for future applications in medical diagnostics, rather than Gemini Advanced. Despite the theoretically superior capabilities of Gemini Advanced [17], Gemini’s framework appears more aligned with practical diagnostic needs and shows greater promise in real-world applications. However, it is essential to verify this trend across a variety of sources to ensure that these findings are not specific to the data sets used in this study. Further investigations involving diverse clinical environments and different types of medical data are crucial to confirm the consistency and reliability of Gemini’s superior performance.

Finally, the comparative analysis of the differentials by Gemini Advanced, Gemini, and Bard revealed consistent inclusion of sepsis, pneumonia, pulmonary embolism, lymphoma, and meningitis among their top 10 differentials. This underscores not only a shared prioritization of these conditions but also the effectiveness of systems in recognizing critical and prevalent diseases. The consistent identification of sepsis, particularly its second-place ranking by Bard, underscores the potential of these AI systems to enhance diagnostic accuracy and reduce errors in the identification of life-threatening conditions [28]. Importantly, the top 3 differentials by Gemini Advanced and Gemini—sepsis, pneumonia, and pulmonary embolism—are among the most harmful diseases where reducing diagnostic errors is crucial [1]. This suggests a potential for GAI systems to alert medical professionals about the inclusion of these important diseases during diagnosis. Such an understanding could facilitate more effective use of these GAI systems in future diagnostics processes.

### Strengths

This study had several strengths. First, the strengths of this study lie in its direct comparison of 3 cutting-edge AI systems and its demonstration of the dynamic improvements in their diagnostic accuracy. Unlike some CDSSs like symptom checkers, whose performance has plateaued [29], these GAI systems evaluated in this research show considerable enhancements with each iteration. Second, we evaluated the diagnostic accuracy of GAI systems using a series of case reports. These case reports often describe rare diseases and atypical presentations, as opposed to common diseases and typical presentations [30]. This showcases the system’s diagnostic capabilities under challenging conditions. Third, the comprehensive range of medical conditions covered by the differential diagnosis lists generated by the AI systems represents a significant strength of this study. This extensive coverage demonstrates the systems’ capacity to handle a broad spectrum of medical knowledge and its applicability to various clinical scenarios.

### Limitations

Several limitations should be discussed. First, the use of case report series might not fully reflect real-world clinical scenarios. This limitation arises because case reports typically focus on novel or rare aspects of diseases rather than typical presentations and common diseases [30]. Second, the exclusive use of a single case report journal could introduce selection bias. Third, there was no well-established method for evaluating AI diagnostics. In our study, we used binary evaluation methods. In contrast, other research on CDSSs used several rating methods [31,32] and the ranking averages in the differential diagnosis lists [33]. Fourth, we used only text data; excluding image data could influence the diagnostic performance. These factors limit the generalizability of these findings.

Concerning the GAI systems, all platforms used in this study were not designed for clinical use and have not received approval for medical diagnostics. These systems were not specifically reinforced or enhanced for medical diagnostic purposes. According to a preprint, Med-Gemini, a specialized model in medicine, was developed [34] but is not available to the public. In addition, we could not include all currently available GAI systems; thus, these findings cannot be generalized to other systems or different clinical scenarios. There was also a risk that these GAI systems may have learned from the published case reports used in this study.

Moreover, the use of user data to refine models, as seen in Gemini Advanced and Gemini, highlights significant privacy concerns [35]. Future research should address the development of locally deployable LLM solutions tailored specifically for CDSS. Although our data set is sourced from an open journal, careful consideration must be given to the ethical deployment of these models within health care settings. Finally, given the rapid pace of GAI technology development, such as the evolution from Bard to Gemini and from ChatGPT-3 to ChatGPT-4 and ChatGPT-4o, our findings may have a limited shelf-life.

### Future Direction

Future research will aim to explore the diagnostic accuracy of GAI systems following medical enhancements and adjustments. Once approved for medical use, it will also be essential to investigate the performance of GAI systems across various populations and settings, including remote medical consultations, to ensure their effectiveness in real-world diagnostics. Moreover, assessing the impact of AI-enhanced diagnostics on the decision-making process of medical professionals will be crucial.

In addition, future studies should focus on integrating GAI systems with existing electronic health record systems to understand how AI can augment data accessibility and analysis. This integration will be essential to evaluate how GAI can improve clinical workflows, reduce the cognitive burden and the time to diagnosis, and enhance patient outcomes.

Finally, the development of ethical guidelines and governance frameworks for the use of GAI in diagnostics is imperative [36]. As AI technologies become more prevalent in health care, it is crucial to establish clear protocols that safeguard patient privacy, ensure data security, and maintain transparency in AI decision-making processes.

### Comparison With Previous Work

Our research builds on previous findings. We revealed that the diagnostic accuracy of ChatGPT-4 was 86.7% (340/392) for the final diagnoses included in the top 10 differential diagnosis lists, and 54.6% (214/392) for the top diagnosis [37]. ChatGPT-4’s performances were still higher than that of Gemini in the lists (76.5% vs 86.7%) and as a top diagnosis (42.6% vs 54.6%); it was similar to Gemini Advanced in the lists (73.0% vs 86.7%) and as a top diagnosis (31.6% vs 54.6%).

Expanding our findings, another study showed that Isabel Pro, a successful CDSS developed by Isabel Healthcare, Ltd [38], correctly identified diagnoses in 87.1% (175/201) of cases, compared with 82.1% (165/201) for ChatGPT-4 in a series of clinical cases [33]. These findings are partly attributed to the earlier launch of Isabel Pro and the ChatGPT series, allowing them to receive more user feedback and undergo updates to improve performance.

In addition, another research focused on multiple choice questions on clinical vignettes revealed that ChatGPT-4 achieved a high accuracy rate of 73.3% for Clinical Challenges from the *Journal of the American Medical Association* (*JAMA*) and 88.7% for Image Challenges from the *New England Journal of Medicine* (*NEJM*). In contrast, Gemini, referred to as Gemini Pro in that study, achieved 63.6% for Clinical Challenges from *JAMA* and 68.7% for Image Challenges from the *NEJM* [22]. While these previous findings and current results revealed certain diagnostic performances of generative AI systems, comparing these results poses significant challenges due to methodological differences. Variations stem from differences in data set preparation, the types of clinical vignettes used, and the specific challenges or images included, which may influence performance outcomes. In addition, the evaluation criteria used to assess accuracy might differ significantly, affecting the comparability. For instance, the scoring systems or the definitions of a “correct” answer could vary, necessitating caution when drawing direct comparisons between these findings and those of this study.

In contrast to the serial evaluation approach of a symptom checker [29], which demonstrated an accuracy of 44.3% (97/219) in the first year and 47.7% (43/90) in the third year without significant difference, the performance of generative AI systems presents a different dynamic. Specifically, the serial evaluation of generative AI indicated that Gemini outperformed Bard over a relatively short period. This superiority can be attributed in part to the adaptability of generative AI systems to incorporate additional data. However, it is crucial to note that this adaptability does not consistently translate into improved diagnostic accuracy, as evidenced by the current comparison between Gemini Advanced and Bard. This observation highlights the nuanced interplay between technological advancement and clinical efficacy, underscoring the need for continued research and validation in integrating these systems into medical practice effectively.

### Conclusions

The results of this study suggest that Gemini outperformed Bard in diagnostic accuracy following the model update. However, Gemini Advanced requires further refinement to optimize its performance for future AI-enhanced diagnostics. These findings should be interpreted cautiously and considered primarily for research purposes, as these GAI systems have not been adjusted for medical diagnostics nor approved for clinical use. The potential and limitations highlighted by this study underscore the need for ongoing development and evaluation of GAI systems within medical diagnostics.

## Appendix

Multimedia Appendix 1 The PubMed search keywords.

Multimedia Appendix 2 The data set of differential diagnosis generated by artificial intelligence systems in this study, alongside the final diagnosis.

## Abbreviations

AI: artificial intelligence
CDSS: clinical decision support system
GAI: generative artificial intelligence
JAMA: Journal of the American Medical Association
LaMDA: language model for dialogue applications
LLM: large language model
NEJM: New England Journal of Medicine
