# Supplementary material for: Comparative Study to Evaluate the Accuracy of Differential Diagnosis Lists Generated by Gemini Advanced, Gemini, and Bard for a Case Report Series Analysis: Cross-Sectional Study
Source: JMIR Med Inform. 2024 Oct 2;12:e63010. doi: 10.2196/63010 (PMC11483254; doi:10.2196/63010)
Supplement: Multimedia Appendix 1 [file medinform_v12i1e63010_app1.docx]

Multimedia Appendix 1. The PubMed search keywords.

| "(2022/1/1:2023/3/1[dp]) AND (American Journal of Case Reports[journal]) |
| --- |
